# Grade III Dermatitis in a Patient Treated With Paclitaxel and Radiotherapy: Case Report and Review of the Literature

**DOI:** 10.4021/wjon314w

**Published:** 2011-06-08

**Authors:** Anna Zygogianni, Konstantinos Gennatas, John Kouvaris, Ioanna Kantzou, Christos Antypas, Maria Tolia, Vassilios Kouloulias

**Affiliations:** a 1st Department of Radiology, Kapodistrian University of Athens, Medical School, Aretaieion Hospital, Greece; b Medical Oncology, Kapodistrian University of Athens, Medical School, Greece; c 1st Department of Radiology, Medical physics section, Kapodistrian University of Athens, Medical School, Greece; d 2nd Department of Radiology, Kapodistrian University of Athens, Medical School, Attikon Hospital, Greece

**Keywords:** Paclitaxel, Taxanes, Radiotherapy, Breast cancer, Case report

## Abstract

Taxanes, both paclitaxel and doxetaxel are the medication of the future in the management of solid tumors. In high risk breast cancer patients, the combination of concurrent paclitaxel and docetaxel chemotherapy with adjuvant radiotherapy is an attractive option to sequential treatment, with potential for enforcing both local and systemic control. This case report examines the tolerance of such treatment. A 54-year-old Greek woman without a relevant medical history, presented with clinical diagnosed breast cancer staged T4NxM0. Neo-adjuvant chemotherapy was initially administered, and paclitaxel was administered concurrently with radiotherapy in order to achieve local control. During the third cycle of paclitaxel the patient developed grade III dermatitis. The tumor showed a reduction in size by 70%, however, chronic cutaneous and subcutaneous changes have not been accessed. In conclusion, adjuvant breast cancer therapy with concurrent standard dose radiotherapy and paclitaxel (175 mg/m^2^) every three weeks, should be approached cautiously owing to paclitaxel induced dermatitis.

## Introduction

Taxanes (both paclitaxel and doxetaxel) are the medications of the future in the management of solid tumors. A trial reported by Sartor [1] showed a survival advantage in node positive women who received sequential paclitaxel in addition to standard doxorubicin and cyclophosphamide chemotherapy. In high risk breast cancer patients, the combination of concurrent paclitaxel and docetaxel chemotherapy with adjuvant radiation is an attractive alternative to sequential treatment, with potential for enhancing both local and systemic control. Although chemotherapy and radiation therapy are very effective modalities against cancer, both of these powerful therapies can have significant adverse effects on oncology patients. This case report examines the tolerance of such treatments.

## Case Report

A 54-year-old Greek woman without a relevant medical history presented with a clinical diagnosis of breast cancer staged T4NxM0 by core needle biopsy. Histology revealed that it was an invasive ductal carcinoma. There were no palpable lymph nodes in the axilla and in the supraclavicular fossa.

The patient was initially administered neoadjuvant chemotherapy with 5-fluorouracil 500 mg/m^2^, adriamycin 50 mg/m^2^ and cyclophosphamide 500 mg/m^2^, showing very little tumor response after the third cycle. The chemotherapy regimen was then changed to gemcitabine 750 mg/m^2^ and cisplatine 30 mg/m^2^ and tumor progression was verified after the fourth cycle. We then opted for a third line of chemotherapy, using paclitaxel 175 mg/m^2^ concurrently with radiotherapy in order to achieve local control.

The patient was treated with a 6 MV linear accelerator. A three dimensional, conformal radiotherapy technique (3DCRT) was used. Patient was treated in the supine position with both arms raised above the shoulder and immobilized. The treatment volume was irradiated by two opposed tangential fields. The medial border was located at the midsternal line. The lateral border was at the midaxillary line to include the breast and to limit the amount of lung at the central plane to less than 2 cm. The superior border was matched at the horizontal line drawn through the supersternal notch, and the inferior border was located at a horizontal line 1.0 - 2.0 cm below the inframammary fold. Wedge compensation was used to ensure a uniform dose distribution throughout the target volume. The dose was prescribed at the isocenter which was placed at point midway along the central plane, two thirds of the distance from the skin to the base of the tangent fields. We kept the dose range between 95% and 107% of prescribed dose. Portal films were obtained in the treatment position with therapeutic beam to confirm patient positioning and adequate coverage.

**Figure 1:**
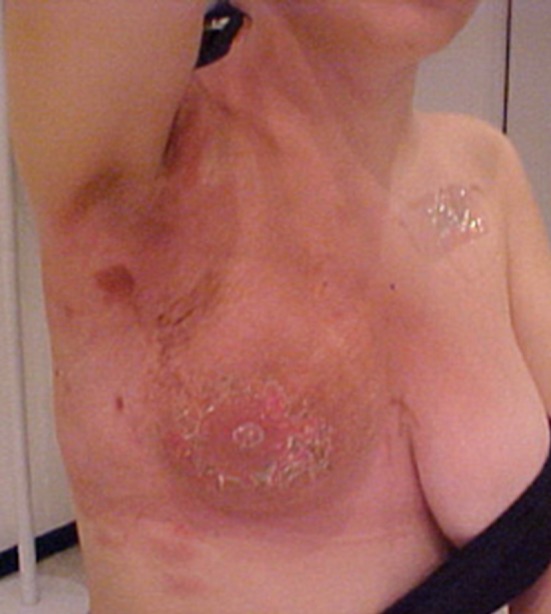
Grade III dermatitis after finishing the radiotherapy treatment.

Daily irradiation dose was 2 Gy in 30 fractions; the total dose was 60 Gy. After finishing the radiotherapy treatment, at third cycle of paclitaxel, the patient developed grade III dermatitis, with intense local hyperchromia and hyperthermia (Fig. 1), followed by continuous dry desquamation throughout the breast. There was a partial response to the therapy and the tumor was reduced by more than 70%. For dermatitis, only topical treatment with corticoid was introduced, producing a gradual improvement of the actinic reaction. After three months of radiotherapy the patient dermatitis improved to grade II. Chronic cutaneous and subcutaneous changes have not been accessed.

## Discussion

Concurrent use of the taxanes and radiation therapy is gaining promise in oncologic management [2]. Bellon et al observed an acceptable rate of local toxicity and a local recurrence rate of 4% with a median follow up of 94 months in 44 patients treated with concurrent use of radiation and taxanes. Grade III acute skin toxicity was seen in 20% of patients. Since that initial experience, the apply of concurrent therapy has been explored, with changes in chemotherapy and dose regimens [3].

A study by Italian researchers found the acceptable combination of capecitabine (CAPE) 1250 mg/m^2^ twice daily with weekly paclitaxel (wPACLI) 80 mg/m^2^ (days 1, 8, and 15) [4]. Severe skin toxicity was not appeared but one event of grade 4 skin rash with the combination of CAPE 1000 mg/m^2^ twice daily and 90 mg/m^2^ wPACLI was reported.

The Swiss Group for Clinical Cancer Research (SAKK) tried to determine the maximum tolerated dose (MTD), toxicity and activity of combined weekly paclitaxel and capecitabine in patients with metastatic breast cancer. Sixteen patients with breast cancer, of whom 15 were evaluable for toxicity and response, were enrolled in 7 Swiss centers [5]. Paclitaxel 80 mg/m^2^ was given intravenously on days 1, 8 and 15. Capecitabine was administered orally on days 1 through 14 using five different dose levels. Both drugs were given in a 21 day schedule. They reported important skin toxicity in 80% of 15 patients such as desquamation. The phase I evaluation of CAPE in combination with fixed dose weekly paclitaxel did not allow the definition of an MTD of cape based on the predefined criteria [5]. Instead, the dose for the phase II evaluation was determined based on the occurrence of toxicity in later courses and on experience with other regimens containing CAPE. They chose CAPE 1000 mg/m^2^ twice daily intermittent schedule and 80 mg/m^2^ wPACLI (days 1, 8, and 15). However, the phase 2 study was closed due to an unfavorable balance between efficacy and predominantly skin toxicity [6].

Bari et al. [7] showed the combination of CAPE 1000 mg/m^2^ twice daily and 60 mg/m^2^ wPACLI (days 1, 8, and 15). Mild and moderate skin rash was observed in 15% of patients. Administration of chemotherapy was delayed in 67% of patients; CAPE dose reductions were required in 64% and PACLI dose reductions in 61% of patients.

Another combination of CAPE 825 mg/m^2^ twice daily and 80 mg/m^2^ of wPACLI (days 1 and 8) was reported by Blum et al. [8]. Skin rash or nail disorders did not occur in their patients. Contrary to these results, the incidence of severe skin adverse events observed in phase 2 studies of the combination of intermittent scheduled CAPE and three weekly PACLI in MBC patients was lower.

Gradishar et al. [9] examined a different combination of CAPE 825 mg/m^2^ and PACLI 175 mg/m^2^. Pharmacokinetic study of CAPE and wPACLI has never been done. Although, a significant clinical interaction between the two agents was not found in two pharmacokinetic analyses of CAPE and three weekly PACLI [10, 11]. There were no crucial differences between plasma area under the curve (AUC) values of CAPE and its metabolites deoxy-fluoro-cytidine, deoxy-fluoro-uridine, and fluorouracil (5FU) in the presence and absence of PACLI. However, the AUC value of fluoro-beta-alanin, the principal 5FU catabolite, was significantly lower in the presence of PACLI. This might suggest that PACLI enhances the peripheral tissue exposure to 5FU when combined with CAPE, possibly caused by PACLI-induced upregulation of TP levels within keratinocytes [10]. This phase II study supports the concept that the complementary mechanisms of action and non-overlapping major toxicities of CAPE and taxanes create a highly effective and well tolerated combination chemotherapy regimen for MBC [11].

The management of unresectable locally advanced breast cancer was studied by Kao et al. Thirty three patients were enrolled as part of two consecutive Phase I/II trials evaluating the safety of concomitant radiation (CRT) and paclitaxel-based chemotherapy between 1995 and 2003. Radiotherapy consisted of 60 - 70 Gy to the breast and chemotherapy consisted of either continuous infusion or bolus paclitaxel. The design of protocols was to determine the escalation of the paclitaxel dose from 5 mg/m^2^ to 20 mg/m^2^. A subset analysis of 16 patients with non-metastatic unresected breast cancer (stage IIIB-C) showed acute toxicity including moist desquamation (n = 8) grade 3 - 4 by the Radiation Therapy Oncology Group (RTOG) scale for acute effects [12].

In addition, 40 patients with operable stage II or III breast cancer received concurrent paclitaxel and radiotherapy were examined by Burstein et al. Paclitaxel was evaluated on 2 schedules, with treatment given either weekly per 12 weeks (60 mg/m^2^), or every 3 weeks per 4 cycles (135 - 175 mg/m^2^). Radiation treatment doses were 39.6 Gy to the breast or 45 Gy to the chest wall. Seven patients developed Grade 2 skin toxicity. There were no cases of Grade 3 or 4 toxicity by RTOG scale [13].

In a trial conducted by Chen et al, 44 women with stage II/III were submitted to breast conserving surgery, 4 cycles of doxorubicin (60 mg/m^2^)/cyclophosphamide (600 mg/m^2^) and 4 cycles of paclitaxel (175 mg/m^2^) delivered every 3 weeks. Radiotherapy was concurrent with the first 2 cycles of paclitaxel. The breast received 39.6 Gy in 22 fractions with a tumor bed boost of 14 Gy in 7 fractions. The 5-year actuarial rate of disease-free survival was 88% and overall survival was 93%. Median follow up was 75 months. Acute skin toxicity grade 3 according to the Radiation Therapy Oncology Group was observed in two patients during the course of radiation therapy. Thirty six patients had complete cosmetic evaluation. Of these, 28 (77.8%) had a mild skin reaction ranging from erythema to dry desquamation, 6 (16.7%) had a small to moderate area of moist desquamation and 2 (5.6%) had a large area of moist desquamation qualifying as grade 3 acute toxicity. No cases of ulceration, hemorrhage, or necrosis of the skin were developed. None of the patients receiving radiation had acute skin toxicity requiring treatment break or delay during radiation [14].

Moreover, a prospective study by William Beaumont Hospital assessed 20 women treated with anthracycline-based adjuvant chemotherapy followed by RT and concurrent paclitaxel (175 mg/m^2^) delivered every 21 days. Patients who underwent breast-conserving therapy and modified radical mastectomy (MRM) were eligible. For patients who were submitted to a breast-conserving therapy, RT consisted of a whole breast dose of 45 Gy with a tumor bed boost to a total of 61 Gy. For patients undergoing MRM, the chest wall received a dose of 50.4 Gy plus a 10-Gy scar boost. It was noticed that radiation pneumonitis developed in 4 patients (20%), and 13 (65%) had Grade 2 cutaneous toxicity or higher [15]. (Table 1)

**Table 1:** Studies of Skin Toxicity From Concurrent Chemotherapy and Radiotherapy

Studies	CAPE dose (mg/m^2^ twice daily from days 1 to 14 every 3 weeks)	Wpacli dose (mg/m^2^, days 1, 8 and 15)	No. of patients	Toxicity (Grade)
Di Constanzo F et al 2006 [4]	1250	80	31	Grade IV: one patient
Unlmann C et al 2005 [5]	1000	80	15	Reported generally
Gick U et al 2006 [6]	825	80	55	NR
Bari M et al 2005 [7]	1000	60	33	Grade I/II: 15%
Blum JL et al 2006 [8]	1000	80	19	NR (non reported)
Gradishar WJ et al 2004 [9]	825	175	47	NR
Kao J et al 2005 [12]	-	Dose escal.: 5 - 20	33	Grade II/IV: 8 patients
Burstein H et al 2006 [13]	-	Weekly: 60 Every 3 weeks: 135 - 175	40	Grade II: 2 patients
Chen W et al 2010 [14]	-	175	44	Grade I: 77.8% Grade II: 16.7% Grade III: 5.6% (2 patients)
Hanna Y et al 2002 [15]	-	175	20	Grade II: 65% (13 patients)

To sum up, more patients and further studies will be required to establish further the safety of concurrent paclitaxel and radiotherapy. Furthermore, the planning of radiotherapy should be more accurate to protect the ipsilateral lung volume and to enable the concurrence with taxanes. Diverse dosing schedules will need to be studied clinically to determine the optimal timing and sequencing of concurrent therapy.

### Conclusion

We have described an unexpected event in the course of chemotherapy and radiotherapy treatment. This event should not be underestimated because of the danger of generalized whole-body skin reaction and the treatment of choice for this should be corticosteroids as soon as the skin reaction is confirmed. We believe that for women receiving adjuvant breast cancer therapy, concurrent standard dose radiotherapy and paclitaxel (175 mg/m^2^) every three weeks should be approached cautiously, because of possible paclitaxel induced dermatitis.
